# Analysis of BRCT5 domain-containing proteins reveals a new component of DNA damage repair in Arabidopsis

**DOI:** 10.3389/fpls.2022.1023358

**Published:** 2022-12-12

**Authors:** Jovanka Vladejić, Fen Yang, Eva Dvořák Tomaštíková, Jaroslav Doležel, Jan J. Palecek, Ales Pecinka

**Affiliations:** ^1^ Institute of Experimental Botany (IEB), Czech Acad Sci, Centre of the Region Haná for Biotechnological and Agricultural Research (CRH), Olomouc, Czechia; ^2^ Department of Cell Biology and Genetics, Faculty of Science, Palacký University, Olomouc, Czechia; ^3^ National Centre for Biomolecular Research (NCBR), Faculty of Science, Masaryk University, Brno, Czechia

**Keywords:** DNA damage repair, genome stability, BRCT domain, BRCT5 domain, homologous recombination, Arabidopsis

## Abstract

The integrity of plant genetic information is constantly challenged by various internal and external factors. Therefore, plants use a sophisticated molecular network to identify, signal and repair damaged DNA. Here, we report on the identification and analysis of four uncharacterized Arabidopsis BRCT5 DOMAIN CONTAINING PROTEINs (BCPs). Proteins with the BRCT5 domain are frequently involved in the maintenance of genome stability across eukaryotes. The screening for sensitivity to induced DNA damage identified BCP1 as the most interesting candidate. We show that BCP1 loss of function mutants are hypersensitive to various types of DNA damage and accumulate an increased number of dead cells in root apical meristems upon DNA damage. Analysis of publicly available *sog1* transcriptomic and SOG1 genome-wide DNA binding data revealed that *BCP1* is inducible by gamma radiation and is a direct target of this key DNA damage signaling transcription factor. Importantly, *bcp1* plants showed a reduced frequency of somatic homologous recombination in response to both endogenous and induced DNA damage. Altogether, we identified a novel plant-specific DNA repair factor that acts downstream of SOG1 in homology-based repair.

## Introduction

Genome stability is constantly threatened by internally and externally-induced DNA damage (Razqallah, 2008; Chatterjee and Walker, 2017). Among others, the presence of damaged DNA negatively affects DNA replication, transcription, and cell cycle progression. Therefore, living organisms developed a sophisticated safeguarding system that recognizes various types of DNA damage, signals their presence, and activates specific molecular effectors that repair the damaged site. This prevents the occurrence of potentially deleterious mutations. Once the repair is completed, the halted cellular processes are restarted and continued. Numerous studies demonstrated that DNA damage repair is essential for the normal growth and fertility of plants, similar to other organisms (Manova and Gruszka, 2015; Nisa et al., 2019) However, despite a generally high degree of evolutionary conservation of the eukaryotic DNA repair system, several unique DNA repair factors evolved in plants (Yoshiyama et al., 2013b; Hu et al., 2016).

Depending on the type of DNA damage, specific DNA repair pathways are activated. A common and highly toxic type of lesion is DNA double-strand break (DSB), which may be generated by external or internal factors. Its persistence in the genome may lead to a loss of genetic information, structural genome changes, and even cell death. The DSB repair begins with a recognition of the damaged site by the MRN (MRE11-RAD50-NBS1) complex and phosphorylation of histone variant H2A.X to produce gamma-H2A.X. This stimulates the binding of the transcription factor Breast cancer type 1 susceptibility protein (BRCA1), followed by signaling through Ataxia Telangiectasia Mutated (ATM) and/or ATM- and RAD3-related (ATR) kinases. The kinase activity of ATM and/or ATR activates the p53 transcription factor at the sites of DNA damage in metazoa and its functional homolog SUPPRESSOR OF GAMMA RADIATION 1 (SOG1) in plants (Preuss and Britt, 2003; Seton-Rogers, 2006; Yoshiyama et al., 2009; Hafner et al., 2019). During the following steps, these transcription factors orchestrate various responses, including pausing of the cell cycle, promotion repair by non-homologous end-joining (NHEJ) or homologous recombination (HR), or (in extreme cases) cell death. In contrast with the error-prone NHEJ, HR represents an error-free mechanism where an intact DNA molecule homologous to the damaged site is used as a template for repair (Heyer et al., 2010). Although the mechanism of HR is studied in great detail across the major branches of the tree of life, not all molecular factors taking place in this process are known.

A prominent group of proteins associated with cell cycle regulation and DNA damage repair contains the BRCA1 C-Terminus (BRCT) domain (Bork et al., 1997), which consists of approximately 100 amino acids and mediates protein-protein interactions by binding to the phosphate groups (Yu et al., 2003). Later studies in animals and yeasts suggested several structurally distinct types of BRCT domains (Wan et al., 2016) The best-studied examples of plant BRCT domain-containing proteins are the BRCA1 and its homolog BREAST CANCER ASSOCIATED RING 1 (BARD1). Both proteins are required for normal levels of somatic HR in plants, and their loss of function mutants are hypersensitive to DNA damage (Trapp et al., 2011). A conspicuous type of BRCT domain is the BRCT5 that was found in budding and fission yeast proteins Rtt107 and Brc1, respectively, and in human protein NSE5/SLF1 (Williams et al., 2010; Li et al., 2012; Räschle et al., 2015). These proteins represent species-specific cofactors involved in the loading of the evolutionary conserved DNA damage repair complex Structural maintenance of chromosomes 5/6 (SMC5/6) to chromatin (Leung et al., 2011; Räschle et al., 2015; Oravcová et al., 2019). However, none of the currently known plant SMC5/6 complex interactors contains this domain. Another example of BRCT5 domain-containing protein includes human Pax2 transactivation domain-interacting protein (PTIP) that performs ATM-dependent activation of p53 and thus promotes DSB repair in mammals (Yan et al., 2011). PTIP also lacks a functional homolog in plants. Therefore, BRCT5 domain proteins represent a little understood group in plants.

Our study demonstrates that analyzing plant proteins carrying BRCT5 domain-containing is an attractive route toward discovering new players involved in the control of plant genome stability. Thus we performed *in silico* identification of Arabidopsis BRCT5 DOMAIN CONTAINING PROTEINs (BCPs). Subsequently, loss of function mutants of four genes was characterized by the expression pattern and hypersensitivity to DNA damage. The most promising candidate BCP1 was analyzed as to its role in HR-based repair.

## Materials and methods

### Plant materials and growth conditions

Unless stated otherwise, all *Arabidopsis thaliana* (Arabidopsis) genotypes used in this study had Columbia (Col-0) background. T-DNA lines used in this study were: GK_301C08 (*bcp1-1*), SALK_001578C (*bcp1-2*), SALK_022790 (*bcp1-3*), GK_076D08 (*bcp2-1*), SALK_111173C (*bcp3-1*), SALK_038422 (*bcp4-1*), and SALK_123114C (*smc6b-1*). T-DNA mutant lines were obtained from the SALK institute (Alonso et al., 2003) and GABI-Kat (Kleinboelting et al., 2012) *via* the European Arabidopsis Stock Centre (NASC). Double mutants were generated by crossing homozygous single mutants and analyzing progeny in F2 generation by PCR-based genotyping for both mutations. HR reporter lines B11 in the C24 background (Swoboda et al., 1994) and IC9C (Puchta et al., 1995; Molinier et al., 2004) were crossed with *bcp1-1*. The resulting hybrids were grown into F4 generation and selected by PCR for double homozygous lines. The oligonucleotides used for genotyping are listed in **Supplementary Table 1**.

Plants used for phenotyping, seed generation, and crossing were grown in climate-controlled phytotron under long-day conditions (at 16 h light, 150 μmol m^−2^ s^−1^ intensity, 19°C during the day; 8 h at 18°C during the night). *In vitro* plant cultivation was done in an air-conditioned phytochamber with a long day regime (16h light, 150 µmol m^-2^ s^-1^, 21°C, 8h dark, 19°C).

### Basic local alignment search tool and BRCT5 domain structure comparisons

New Arabidopsis BRCT5 domain-containing proteins were identified by the BLAST search (Altschul et al., 1990) using the fission yeast *Schizosaccharomyces pombe* Brc1 (*Sp*Brc1) and human *Homo sapiens* NSE5 (*Hs*NSE5) proteins against the *Arabidopsis thaliana* database (taxid: 3702). The retrieved BRCT5 domain sequences were manually aligned, and their AlphaFold structural models (Varadi et al., 2022) were compared.

### Molecular cloning, plant transformation, and GUS assays

To develop the promoter-reporter line, a region 2000 bp upstream of the *BCP1* transcription start site (*ProBCP1*) was amplified by PCR and cloned by Gateway Technology (ThermoFisher Scientific; Cat. nos.: 11789100, 12538120) into *pDONR207* (Invitrogen) and then recombined into the binary vector *pKGWFS7.0*, containing *uidA* gene encoding β-glucuronidase (GUS). Plasmids carrying the *ProBCP1::GUS* fusion were transformed into *Agrobacterium tumefaciens* strain GV3101. Transformation of *Arabidopsis* Col-0 was performed using the floral dip method. (Zhang et al., 2006). The selection of transformed plants in T1 generation was carried out on a medium containing 100 μg/ml kanamycin (Sigma-Aldrich, Cat. no. 60615). Resistant plants were transferred to soil for seed production. The following generation (T2) of plants was selected based on the activation of the *GUS* reporter gene. The oligonucleotides used for genotyping or cloning are listed in **Supplementary Table 1**.

The expression pattern of *BCP1* in highly dividing vegetative tissues, as well as the change of expression under DNA damage stress, was examined by using a *ProBCP1::GUS* reporter line. Plants grown for seven days on a solid medium were transferred for 24 h to liquid ½ MS medium with or without 10 μM mitomycin C (MMC, Cat. no. M0503), a genotoxic agent causing intrastrand DNA crosslinks. Following the treatments, plants were stained by GUS histochemical staining. GUS solution containing 10 mM EDTA (Sigma-Aldrich, Cat. no. E5134), 2 mM potassium ferrocyanide (Lachema, Cat. no. 68 4514), 2 mM potassium ferricyanide (Lachema), 100 mM disodium phosphate (Penta, Cat. no. 15150), 100 mM monosodium phosphate (Lachema, Cat. no. 68 4639), 0.1% Triton X-100 (Sigma-Aldrich, Cat. no. T8787) and 2 mM X-Gluc (Thermo Scientific, Cat. no. R0852) was prepared as described in (Baubec et al., 2009). Seedlings were transferred to 5 ml tubes and infiltrated with GUS staining solution under a vacuum. After five to ten minutes, the vacuum was released and tubes were placed at 37°C overnight. Subsequently, the GUS staining solution was removed and plants were cleared by incubation in 70% ethanol (v/v) at 37°C. Ethanol was changed 3 times, and after the last change, plants were left overnight at 4°C. Pictures were taken under a stereo-microscope (Olympus SZX16) and fluorescent microscope (Olympus BX60).

For the analysis of *BCP1* expression in the reproductive tissues, inflorescences were fixed in 90% (v/v) acetone and incubated for 45 min at -20°C. Acetone was then removed, and samples were washed three times with 100 mM phosphate buffer, pH 7.2. After washing, the flowers were infiltrated with GUS staining solution under a vacuum for 10 min and left overnight at 37°C. The next morning the solution was removed, and samples were washed shortly with phosphate buffer and cleared in chloral hydrate solution containing eight parts chloral hydrate (Sigma-Aldrich, Cat. no. 23100) two parts water, and one part glycerol (Sigma-Aldrich, Cat. no. G516). Flowers were mounted on the microscope slide and dissected in the same solution. Pictures were taken under a stereo-microscope (Olympus SZX16) and fluorescent microscope (Olympus BX60).

### Root sensitivity assays

For root sensitivity assays, surface sterilized and stratified seeds were grown on ½ MS growth medium with 0.6% agar (w/v) and 1% sucrose (w/v). Seeds were sterilized in 70% ethanol (v/v) for 5 min, followed by 8% sodium hypochlorite solution (v/v) for 6-10 min, and washed 3 times in sterile water. Seeds were stratified for 48h in 0.1% agarose solution (w/v) at 4°C in the dark. Stratified seeds were evenly distributed on Petri dishes containing ½ MS medium (mock) or ½ MS medium supplemented with 10 μM MMC (Sigma-Aldrich, Cat. no. M0503), 20 nM camptothecin (CPT; Sigma-Aldrich, Cat. no. C9911), 20 μM zebularine (Sigma-Aldrich, Cat. no. Z4775), or 50 nM bleomycin (Sigma-Aldrich, Cat. no. 203408-M). Plants grown for seven days in a horizontal position were then carefully pulled off the medium using tweezers and laid flat on a plate with agar. The length of the primary root was measured using the ImageJ plugin SmartRoot (Lobet et al., 2011). Experiments were performed in three biological replicates with typically 20 plants per replicate (minimum of 11 plants in one replicate). Statistical significance was tested with One-way ANOVA with posthoc Tukey HSD in Minitab.

### Cell death assays

Sterilized and stratified seeds were grown vertically on plates with ½ MS medium with 0.8% agar (w/v) for five days and then transferred into liquid ½ MS medium for a 24 h treatment. Mock samples were grown in pure liquid ½ MS medium, while treated plants had medium supplemented with 10 μM MMC. Following the treatment, seedlings were stained with 10 mg.mL^-1^ propidium iodide solution (Sigma) on glass microscope slides. Visualization and photography were performed using Leica confocal microscope TCS SP8 (Leica, Wetzlar, Germany) and HC PL APO CS2 20x/0.75 DRY objective equipped with Leica LAS-X software with Leica Lightning module laser scanning confocal microscope (Leica). At least 13 plants for each group were analyzed. The means of the three replicates are depicted. Statistical significance was tested withKruskall-Wallis H-test with *post hoc* Conover-Iman test of multiple comparisons using rank sums with Benjamini-Hochberg procedure in R 4.2.1 (R Core Team, 2018).

### Homologous recombination assays

The B11, B11 *bcp1-1*, IC9C, and IC9C *bcp1-1* plants were grown on ½ MS medium with or without (mock) 1 μM MMC under sterile conditions. Ten days-old seedlings were histochemically stained using GUS as described above. Plants were transferred to a Petri dish containing ethanol and examined using a stereo-microscope (Olympus SZX16) for HR events identified as blue-stained cells or areas. The means of the three replicates are depicted. Statistical significance was tested withMann-Whitney U-tes in Minitab (www.minitab.com).

### Fresh weight measurements

Plants were grown as described in homologous recombination assays were measured on an analytical scale. Measuring was done in triplicates, and each sample was composed of 60 seedlings. Mann-Whitney U-test (P < 0.05) was used to assess the significance of weight differences (www.minitab.com).

### RNA-seq data analysis

RNA-seq data for wild-type and *sog1-1* plants were obtained from a publicly available dataset (Bourbousse et al., 2018). The database contains gene expression values (fragment per kilobase per million reads, FPKM) in plants grown under normal and DNA damaging conditions at six-time points post gamma irradiation (20 min, 1.5, 3, 6, 12, and 24 h). From it, we acquired expression profiles of BCPs. The changes in gene expression were assessed as described in the results. For the assessment of statistical significance, we used a two-sample T-test with unequal variances.

### Reverse transcription-quantitative polymerase chain reaction

T-DNA mutants lines’ seeds, sterilized and stratified, were grown on ½ MS medium with 0.6% agarose. Seven days old seedlings were sampled and flash-frozen in liquid nitrogen. RNA extraction was performed by RNeasy Mini Kit (Qiagen, Cat. no. 74104). cDNA was constructed with RevertAid H Minus First Strand cDNA Synthesis Kit (Thermo Scientific™, Cat. no. K1631). The qPCR was performed with the HOT FIREPol^®^ EvaGreen^®^ qPCR Mix Plus (Solis BioDyne, Cat. no. 08-24-0000S) in CFX96 Touch Real-Time PCR Detection System. Wild type and *sog1-1* plants were grown for seven days in ½ MS 0.6% agarose and then transferred to liquid ½ MS with or without 40 μM MMC for a 1 h treatment. Following the treatment samples were flash-frozen in liquid nitrogen, and RNA extraction, cDNA synthesis and RT-qPCR were performed as described above. Mann-Whitney U-test was performed in Minitab to assess statistical significance of the data.

### Accession numbers

Gene information and sequences used in this article can be found in TAIR under the following accession numbers: *BCP1* (AT4G02110), *BCP2* (AT2G41450), *BCP3* (AT4G03130), *BCP4* (AT3G21480), *SMC6B* (AT5G61460).

## Results

### Identification of Arabidopsis BRCT5 domain-containing proteins

**Figure 1:**
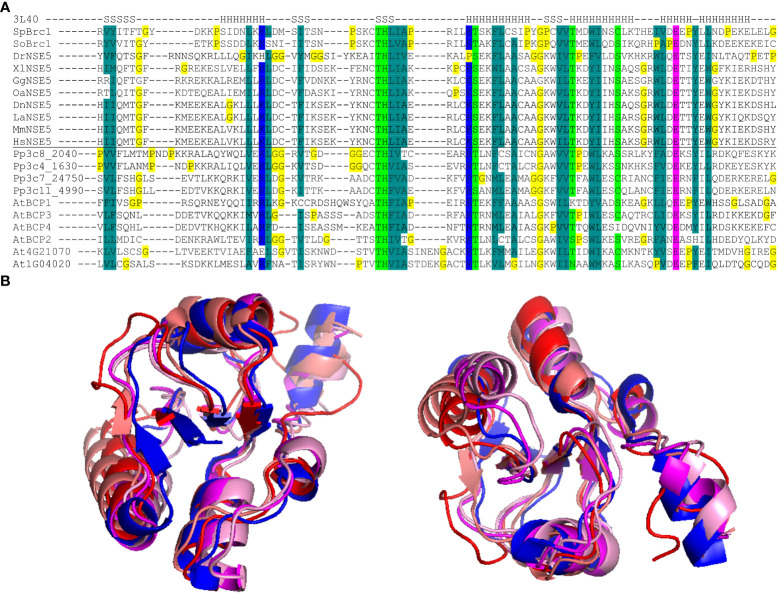
BRCT5 domain analysis. **(A)** Alignment of the core part of the BRCT5 domain with helical (H) and β-strand (S) segments above (from PDB: 3L40 structure; Williams et al., 2010). The Brc1 and NSE5 orthologs are from *Schizosaccharomyces pombe* (*Sp*), *S. octosporus* (*So*), *Physcomitrium patens* (*Pp*), *Arabidopsis thaliana* (*At*), *Danio rerio (Dr), Xenopus laevis* (*Xl*), *Gallus gallus* (*Gg*), *Ornithorhynchus anatinus* (*Oa*), *Monodelphis domestica* (*Md*), *Dasypus novemcinctus* (*Dn*), *Loxodonta africana (La), Mus musculus* (*Mm*), *Homo sapiens* (*Hs*). Coloring indicates amino acid groups conserved across the family: *dark green*, hydrophobic and aromatic; *light green*, polar; *blue*, acidic; *pink*, basic; all glycine and proline residues are highlighted in *yellow*. **(B)** Superimposition of modeled BRCT5 domains of At4g02110 (red), At2g41450 (deep pink), At4g03130 (coral), At3g21480 (pale pink), and crystal structure *Sp*Brc1 (blue) shown from two views.

To identify potential Arabidopsis BRCT5 domain-containing proteins, we performed a BLAST search (Altschul et al., 1990) using the BRCT5 domains of the fission yeast *Schizosaccharomyces pombe* Brc1 (*Sp*Brc1) and human *Homo sapiens* NSE5 (*Hs*NSE5) proteins against the Arabidopsis protein database. Three candidates, At4g02110, At4g03130, and At4G21070, were found as potential genes of interest. We hypothesized that the large phylogenetic distance between Arabidopsis versus yeast and human might have reduced the efficiency of such a screen and compromised the direct identification of some candidates. Therefore, we performed an additional search for the BRCT5 domain-containing proteins in the genome of moss *Physcomitrium patens* using the same query sequences from *Sp*Brc1 and *Hs*NSE5. The BRCT5 domains were found in the moss proteins Pp3c4_1630, Pp3c7_24750, Pp3c8_2040, and Pp3c11_4990. As the next step, the moss proteins were BLASTed against the Arabidopsis genome, which revealed three additional genes At1g04020, At2g41450, and At3g21480. The sequence comparison identified a conserved pattern of amino acids (**Figure 1A**) with different properties typical for the BRCT5 type domain that supported all the candidates identified *via* BLAST. The candidates At4g21070 and At1g04020 were previously described as *Arabidopsis* orthologs of human *BREAST CANCER SUSCEPTIBILITY 1* (*BRCA1*) and its homolog *BREAST CANCER ASSOCIATED RING 1* (*BARD1*), whose functions in plant DNA damage repair have been already documented (Lafarge and Montané, 2003; Reidt et al., 2006). Therefore, both *BRCA1* and *BARD1* were excluded from subsequent analyses. Based on this, we selected the remaining four candidate proteins for further analysis and named them BRCT5 DOMAIN-CONTAINING PROTEINs (BCPs): BCP1 (At4g02110), BCP2 (At2g41450), BCP3 (At4g03130) and BCP4 (At3g21480). The superimposition of computationally modeled BRCT5 domains of *Sp*Brc1 and the four selected BCPs revealed their high structural similarity (**Figure 1B**). Based on Araport11 gene annotation, *BCP1* is a cell cycle regulated transcriptional coactivator (Menges et al., 2002). Based on its two BRCT domains, it was also considered a possible candidate for plant homolog of the human DNA topoisomerase 2-binding protein 1 (TOPBP1), but the overall low sequence similarity did not allow for the drawing of a firm conclusion (Shultz et al., 2007). *BCP2* is described as N-acetyltransferase (Araport11) and is expressed in the female gametophyte (Wuest et al., 2010). *BCP3* and *BCP4* are both described as BRCT domain-containing DNA repair proteins (Araport11), likely based on the presence of their C-terminal BRCT domains, with no further information. Hence, we identified six BRCT5 domain-containing candidates in Arabidopsis, with four of them representing uncharacterized Arabidopsis genes.

### Loss of *BCP1* causes sensitivity to DNA damage, and its transcription is SOG1 dependent

**Figure 2:**
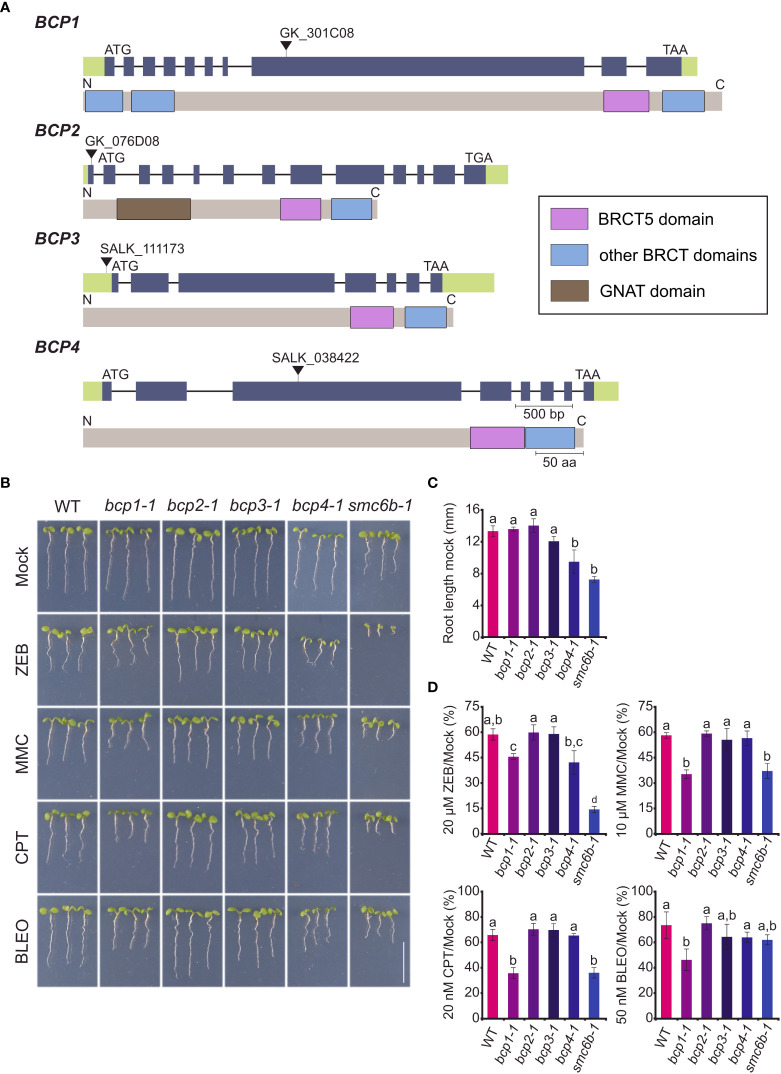
Phenotypes of mutants in uncharacterized Arabidopsis BRCT5 domain-containing genes. **(A)** Gene and protein structures of the BRCT5 CONTAINING PROTEINS (BCPs). The positions of T-DNAs in the used mutant alleles are indicated by black triangles above the gene models. Introns are indicated by a horizontal line and exons by green (untranslated regions) and purple (coding sequence) colors. Protein models under gene models (grey) show the position of known domains: BRCT - blue rectangles and GNAT (Gcn5-related N-acetyltransferase) - brown rectangle. **(B)** Representative phenotypes of seven days old wild-type (WT) and homozygous mutant plants grown on media containing 20 μM zebularine (ZEB), 10 μM mitomycin C (MMC), 20 nM camptothecin (CPT), 50nM nM bleocin (BLEO). The *smc6b-1* served as a sensitive control. Scale bar = 1 cm. **(C)** Root length of WT and mutant plants under control (mock) conditions. Error bars indicate the standard deviation between the means of three biological replicates. The letters above columns indicate similarities between samples. The same letters indicate samples that were not significantly different in one-way ANOVA with *post hoc* Tukey’s test (*P* < 0.05). **(D)** Root length of WT and mutants from B under DNA damaging treatments relative to the growth of the same genotype under mock conditions. Error bars represent the standard deviation between three biological replicates, each with at least 15 plants. Statistics were performed as in **(C)**.

To test for the potential role of *BCPs* in DNA damage repair, we isolated their T-DNA insertional mutants (**Figure 2A**). All homozygous mutants were viable and did not show any obvious developmental defects during somatic development and/or sterility during the reproductive stage.

As the first step, we performed an initial screening for mutant sensitivity to different types of DNA damage. The aim was to identify if any of the genes are important for DNA damage repair. To this end, we focused on the induction of all possible types of DNA damage including DSBs, DNA inter-strand, and DNA-protein crosslinks. Seeds were germinated, plants were grown on the genotoxin-containing media, and their root length was measured (**Figure 2B**). The *smc6b-1* mutant allele of *STRUCTURAL MAINTENANCE OF CHROMOSOMES 6B* (*SMC6B*) served as a hypersensitive control. Under mock conditions, only the *bcp4-1* mutant plants had significantly shorter roots (9.4 mm ± 1.5 mm in *bcp1-4* compared to 13.23 mm ± 0.68 mm in WT control), while the root length of the remaining mutants was not significantly different from the wild type (**Figure 2C**). Under DNA damaging conditions, the *bcp1-1* plants were hypersensitive to 10 μM DNA inter-strand cross-linker MMC, 20 nM DNA-protein cross-linker CPT and 20 nM radiomimetic agent bleomycin causing DNA strand breaks. The *bcp1-1* plants also exhibited sensitivity to 20 μM type I DNA-protein cross-linker zebularine (Prochazkova et al., 2022). The *bcp2-1*, *bcp3-1*, and *bcp4-1* mutant plants did not show significantly increased sensitivity to any of the genotoxic treatments (**Figure 2D**).

Absence of sensitivity in combination with non-coding sequence location of T-DNAs stimulated us to analyze the expression of BCPs in their corresponding T-DNA insertion mutant lines by RT-qPCR (**Supplementary Figure 1**). The *bcp1-1* and *bcp4-1* showed a very strongly reduced amount of transcript compared to their WT variants. Surprisingly, the *bcp2-1* with T-DNA insertion in the first out of total 13 exons showed more than 90-fold over-expression of *BCP2*. This might be caused by the expression from the *Cauliflower Mosaic Virus 35S* promoter that is part of the T-DNA insertion. The *bcp3-1* showed no significant difference in the amount of transcript compared to wild type. This suggests that *bcp1-1* and *bcp4-1* are loss of function mutants, *bcp2-1* is a potential overexpressor line and *bcp3-1* might not affect BCP3 gene function.

**Figure 3:**
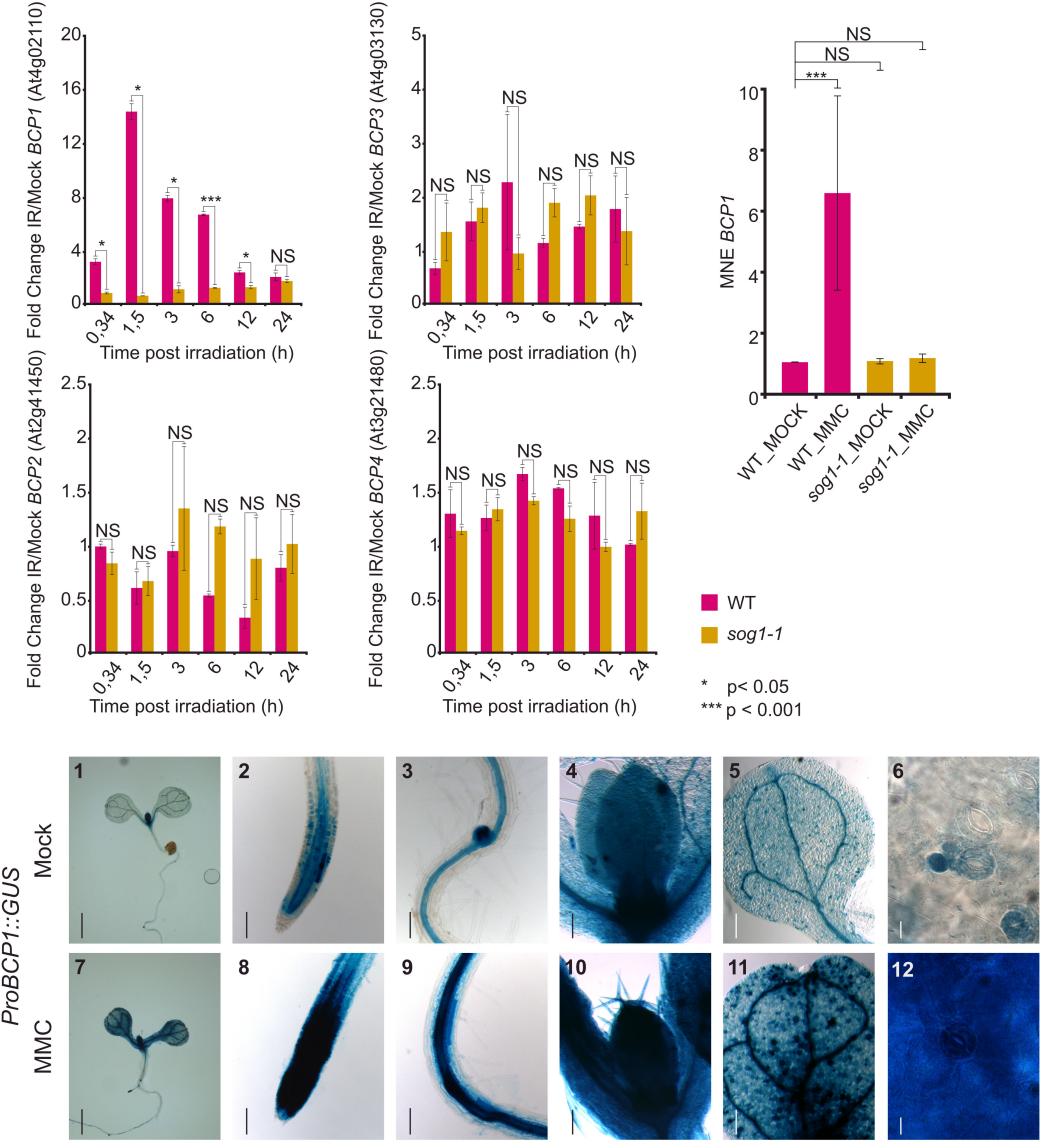
Expression of the *BCP* genes. **(A)** Relative expression of *BCP1* to *BCP4* in wild type (WT) and *sog1-1* based on RNA-sequencing experiment of Bourbousse et al. (2018). The normalized read counts from RNA-sequencing (FPKM) were used to calculate fold change in expression after gamma irradiation pulse (IR) versus mock conditions (y-axis). The x-axis indicates the harvesting time after the irradiation treatment. Error bars represent the standard error of the mean. Asterisks represent significant differences in two sample T-test with unequal variance, * *P <* 0.05, *** P < 0.001, NS – not significantly different. **(B)** Reverse transcription qPCR analysis of *BCP1* expression in wild-type (WT) and *sog1-1* plants without (MOCK) and after 1 h treatment with 40 μM MMC (MMC). Y-axis shows mean normalized expression relative to *PP2A*. Error bars show three biological replicates. NS = not significantly different, *** statistically significantly different in Mann-Whitney U-test at *P* < 0.05. **(C)** *In planta* analysis of *BCP1* promoter activity. Seven days old seedlings carrying *ProBCP1::GUS* were transferred to mock and DNA damaging conditions for 24h. *BCP1* promoter activity was monitored using GUS histochemical staining. Representative stereo microscope pictures of tissues showing the gene expression. (1,7) Whole seedling, (2, 8) shoot apical meristem, (3, 9) lateral root meristem, (4, 10) shoot apical meristem with first real leaves, (5, 11) cotyledon, (6, 12) leaf blade cells. Scale bars: 1,7 = 1 mm; 2-5,8-11 = 100 μm, 6,12 = 50 μm.

Next, we analyzed the expression of the BCP candidates using available transcriptomic data. Surprisingly, none of the selected candidates is represented on the Arabidopsis ATH1 expression array. RNA-sequencing-based atlas of Arabidopsis developmental stages (Klepikova et al., 2016) revealed that *BCP2* and *BCP3* were only weakly expressed throughout the whole plant development and that the expression slightly increased only in some floral parts (**Supplementary Figure 2**). In contrast, both *BCP1* and *BCP4* showed a low to moderate expression with the highest values observed in floral organs and seeds. Surprisingly, only weak expression was found in the root tissues. To find a potential involvement of *BCPs* in DNA damage response, we analyzed their expression after gamma-irradiation in wild-type and *sog1* mutant background using a publicly available RNA-seq dataset (Bourbousse et al., 2018). Under ambient (mock) conditions, *BCP1* was expressed stably at a basal level in both WT and *sog1-1* plants (**Supplementary Figure 3A**). In response to gamma-irradiation, *BCP1* was upregulated 3.2-fold already 20 min post-treatment, and the amount of transcript reached its 14-fold increase maximum 1.5 h post-irradiation (**Figure 3A**). The amount of transcript lowered over time and returned to mock levels 24 h after the treatment. In the *sog1* plants, gamma radiation-induced expression was not observed, suggesting that transcriptional response of *BCP1* to DNA damage is SOG1-dependent, and that *BCP1* acts downstream of SOG1. This was confirmed also in RT-qPCR experiment where *BCP1* was significantly up-regulated in response to MMC treatment in wild-type but not in *sog1-1* mutant plants (**Figure 3B**). However, the same amount of *BCP1* expression in mock-treated wild-type and *sog1-1* plants indicates that basal *BCP1* expression is SOG1 independent. The remaining genes *BCP2*, *BCP3*, and *BCP4* showed only minor transcriptional changes that differed between wild-type and mutant plants, mostly at solitary time-points, suggesting that these genes are not gamma-irradiation inducible and their expression is not SOG1*-*dependent (**Figure 3A**).

To gain more insights into the transcriptional response of *BCP1* to DNA damage, we generated stable Arabidopsis transformants carrying *BCP1* promoter fused with the *GUS* reporter gene (*ProBCP1::GUS*). Under mock conditions, the *BCP1* promoter was active in tissues with actively dividing cells, such as the root and shoot apical meristems, lateral root meristems, and vasculature (**Figure 3C**_2,3,4). The signals in true leaves had a peculiar dotted pattern. After inspection at the cellular level, it was obvious that these “dots” are represented by young stomata guard cells, stomatal lineage ground cells, and guard mother cells. The older (larger) stomata guard cells and pavement cells showed little or no GUS signals (**Figure 3C**_5,6). As the Arabidopsis transcriptomic atlas (Klepikova et al., 2016) data suggested the highest *BCP1* transcript amount in reproduction (**Supplementary Figure 2**), we further examined the pattern of *BCP1* expression in inflorescences (**Supplementary Figures 4A–E**). We found particularly strong GUS signals in pistils throughout the entire flower development, young stamen, filaments, and perianth of closed flowers.

To visually confirm that the *BPC1* transcription is induced by DNA damage, as suggested by the transcriptomic data, we exposed seedlings of the *ProBCP1::GUS* reporter line to 10 μM MMC for 24 h and subsequently scored *BCP1* promoter activity. Intense signals appeared in almost all parts of the plant, including the true leaves (**Figure 3C**_7-12). This strongly supports transcriptomic data and demonstrates that BCP1 transcription is inducible by DNA damage. Based on these experiments, we considered *BCP1* as the most promising candidate for further analysis.

### *BCP1* is required for the repair of various types of DNA damage

**Figure 4:**
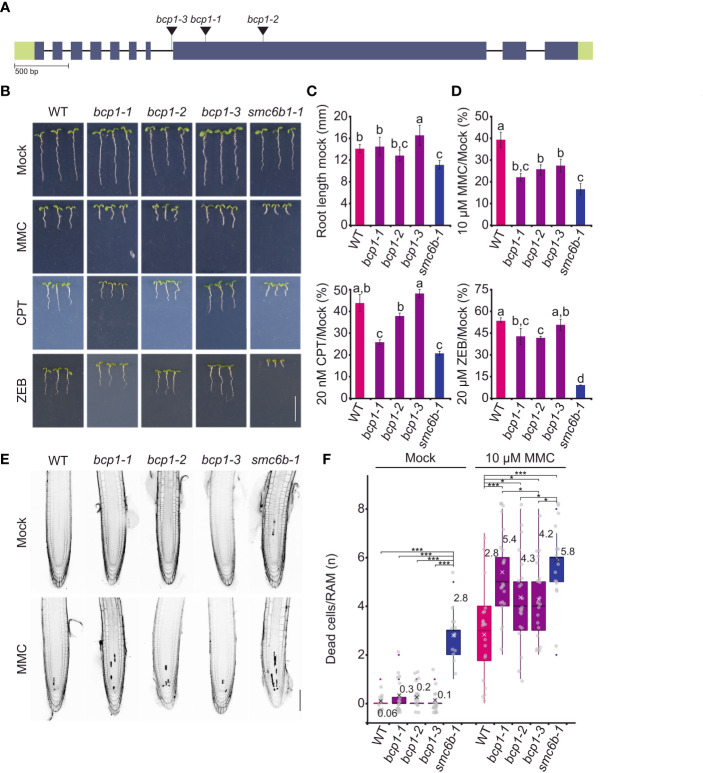
BCP1 is required for normal resistance to DNA damaging treatments. **(A)** Gene model of BCP1 with indicated positions of all used T-DNA insertional mutants. The style follows the description in **Figure 2A**. **(B)** DNA damage sensitivity assays of different bcp1 alleles. Representative phenotypes of seven days old wild-type (WT) and homozygous mutant plants grown on media containing 20 μM zebularine (ZEB), 10 μM mitomycin C (MMC), and 20 nM camptothecin (CPT). The smc6b-1 served as a sensitive control. Scale bar = 1 cm. **(C)** Root length of WT and mutant plants grown under control (mock) conditions. Error bars indicate the standard deviation between the means of nine biological replicates. The letters above columns indicate similarity between samples. The same letters indicate samples that were not significantly different in one-way ANOVA with posthoc Tukey’s test (P < 0.05). **(D)** Root length of WT and mutants under DNA damaging treatments relative to the growth of the same genotypes under mock conditions. Statistics were performed as in **(C)**. **(E)** Representative confocal microscopy images of the primary roots stained by propidium iodide in cell death assays. Five days old seedlings of WT and bcp1 mutants were exposed to mock or 10 μM MMC treatments for 24 h, stained with propidium iodide, and analyzed to reveal dead cells that appear as dark sectors inside the roots. The smc6b-1 served as a control with increased cell death. Scale bar = 100 μm. **(F)** Quantification of dead cells per root apical meristem in different genotypes and treatments (complements E). Each gray dot indicates the number of dead cells per root (n = 13-22). The boxplots’ hinges are in the 1st and 3rd quartile, with a marked median. The mean is indicated by a cross with a numerical value. Whisker marks show the lowest or highest value within the 1.5 interquartile range below or above hinges. Statistical significance was tested by Kruskall-Wallis H-test with post hoc Conover-Iman test of multiple comparisons with Benjamini-Hochberg procedure (*P* < ½ α, α = 0.05). NS - not significant, * *P* < 0.025, *** *P* < 0.001.

To validate our initial findings based on a single mutant allele, we isolated two more *BCP1* T-DNA-insertional mutants located in the 8^th^ exon (*bcp1-2*) and the 7^th^ intron (*bcp1-3*) (**Figure 4A**). Phenotypic analysis of all three homozygous mutant lines confirmed the absence of obvious developmental defects at four and six weeks of age (**Supplementary Figure 5**).

Next, we extended the sensitivity assays by exposing plants of all *bcp1* mutant lines to 10 μM MMC, 20 μM zebularine, and 20 nM CPT (**Figures 4B, D**). In mock conditions *bcp1-1* and *bcp1-2* mutants showed no difference in root length compared to wild type, while *bcp1-3* plants had slightly longer roots (**Figure 4C**). Both *bcp1-1 and bcp1-2* alleles were significantly sensitive to all three drug treatments. In contrast, the intronic mutant *bcp1-3* was hypersensitive only to 10 μM MMC (**Figure 4D**). This is in agreement with the amount of *BCP1* transcript which was almost not detectable in *bcp1-1* and *bcp1-2* mutants but was not significantly reduced in *bcp3-1* (**Supplementary Figure 1**). Specifically, MMC-treated wild-type plants reached 38.8 ± 3.6% of the standard root length compared to mock conditions, while it was only 21.8 ± 1.7%, 25.5 ± 2.5%, and 27 ± 3% of the mock-treated plant root length for *bcp1-1*, *bcp1-2*, and *bcp1-3*, respectively (all comparisons *P <*0.001 one-way ANOVA with *post hoc* Tukey HSD). Zebularine-treated wild-type plants reached 53.3 ± 1.7% of the mock control length. For zebularine treated *bcp1-1*, *bcp1-2* and *bcp1-3* plants it was 42.7 ± 5.6%, 41.6 ± 1.25% and 50.5. ± 4.0%, respectively (all comparisons *P <*0.001). Similarly, CPT-treated wild-type plants reached 43.2 ± 4% of the normal root length, but it was 25.5 ± 1.1%, 37.4 ± 1.4%, and 47.5 ± 2% for the individual *bcp1* mutant alleles, respectively (all comparisons *P <*0.001 one-way ANOVA with *post hoc* Tukey HSD). The sensitive control *smc6b-1* plants had massive root length reduction to 16 ± 2%, 10 ± 0.3%, and 20 ± 1% for MMC, zebularine, and CPT (all comparisons *P <*0.001 one-way ANOVA with *post hoc* Tukey HSD).

To assess the extent of damage at the cellular level, we performed a cell death assay based on the staining of root apices with propidium iodide (PI), where PI marks the dead cells and is excluded from the living cells (**Figures 4E, F**). Wild-type and *smc6b-1* were used as standard and hypersensitive controls. Mock-treated wild-type and *bcp1* plants showed no significantly different mean values of less than one dead cell per root (**Figures 4E, F**). After the treatment with 10 μM MMC for 24 h, the median number of dead cells per root increased to three in wild type, four in *bcp1-2* and *bcp1-3*, and five in *bcp1-1* (**Figure 4F**). The values in all mutant lines were significantly higher compared to wild-type plants. This shows that loss of function from *BCP1* makes Arabidopsis plants hypersensitive to diverse types of DNA damage and leads to increased cell death.

### BCP1 is required for normal frequency of homologous recombination

**Figure 5:**
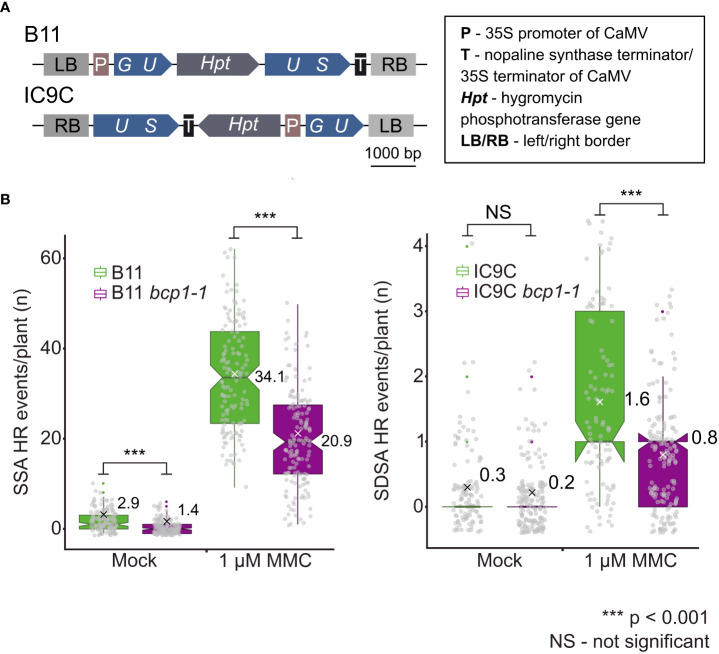
**(A)** Schematic model of constructs used to create *B11* and *IC9C* HR reporter lines (Swoboda et al., 1994; Molinier et al., 2004). **(B)** Loss of BCP1 causes reduced frequency of somatic homologous recombination (HR). Wild type (WT) and *bcp1-1* plants carrying genomic substrates for single-strand annealing (*B11*) and synthesis-dependent strand annealing (*IC9C*) types of HR were grown on mock and 1 μM containing MMC media for 10 days. Gray dots indicate HR events per plant. The boxplots’ hinges are in the 1^st^ and 3^rd^ quartile, with a marked median. Mean is represented by a cross with a numerical value. Whisker marks show the lowest or highest value within the 1.5 interquartile range below or above hinges. Asterisks represent significant differences in Mann-Whitney U-test *** P < 0.001, NS – not significantly different.

Based on the SOG1*-*dependent transcriptional activation of *BCP1* upon DNA damage and hypersensitivity of *bcp1* plants to DNA damaging treatments, we hypothesized about a possible role of BCP1 in HR. To experimentally test this hypothesis, we generated double homozygous *bcp1-1 B11* and *bcp1-1 IC9C* lines (Swoboda et al., 1994; Puchta et al., 1995; Molinier et al., 2004). Owing to the organization of the reporter regions, these lines allow locus-specific monitoring of the frequency of single-strand annealing (SSA) and synthesis-dependent strand annealing (SDSA) types of HR, respectively (Orel et al., 2003). The plants were germinated and grown on media without (mock) and with 1 μM MMC for 10 days and analyzed for HR events. There were no significant differences (Mann-Whitney U test, P > 0.05) in fresh weight between all genotypes (**Supplementary Figure 6**), indicating similar number of cells. Under mock conditions, we found on average 2.9 ± 2.2 SSA HR events per *B11* wild type (n = 131) plant (**Figure 5**), while *bcp1-1 B11* plants (n = 151) showed 52% less SSA HR events per plant (1.4 ± 1.5). This difference was statistically significantly different (*P* < 0.001 Mann-Whitney U-test), indicating a possible role of *BCP1* in HR independent of exogenous DNA damage. In response to a mild DNA inter-strand crosslinking treatment by MMC, there were on average 34 ± 12 SSA HR events per *B11* plant (n = 137 plants) and 21 ± 10.6 per *bcp1-1 B11* plant (n = 151 plants), corresponding to a significant (*P* < 0.001Mann-Whitney U-test) 38% reduction in the mutant.

A similar pattern was observed in SDSA HR reporter line IC9C. Also here, we did not find significant differences (Mann-Whitney U-test, P > 0.05) in fresh weight between all genotypes (**Supplementary Figure 6**), indicating similar number of cells. The IC9C wild type and IC9C *bcp1-1* lines showed a similar 0.2 ± 0.46 (n = 153 plants) and 0.28 ± 0.61 (n = 109 plants) SDSA events per plant under mock conditions, respectively. After treatment with 1 µM MMC, IC9C wild type showed an average of 1.59 ± 1.31 (n = 102 plants) SDSA HR events plant, while IC9C *bcp1-1* line had 0.78 ± 0.85 (n = 158 plants) SDSA HR events per plant. This is a 50.5% decrease in the number of HR events in the *bcp1-1* mutant under mild genotoxic stress. Collectively, this shows that BCP1 is needed for normal levels of SSA and SDSA HR in Arabidopsis and suggests an involvement of BCP1 in the HR repair.

## Discussion

In this work, we found a new Arabidopsis protein BCP1 which contains the BRCT5 domain and contributes to DNA damage repair by homologous recombination in a SOG1-dependent manner.

To identify Arabidopsis BRCT5 domain-containing proteins, we performed a homology search using fission yeast *Sp*Brc1 and human *Hs*NSE5. These proteins were selected because they are known to mediate interactions of the conserved SMC5/6 DNA repair complex to chromatin (Li et al., 2012; Räschle et al., 2015). While human NSE5 directly contains the BRCT5 domain, the yeast NSE5 does not, but it binds BRCT5-containing Brc1 protein which targets it to DNA damage sites. The situation in Arabidopsis resembles yeast where none of the currently known SMC5/6 complex subunits harbors a BRCT domain (Yan et al., 2013). Hence, identification of the plant BRCT5 domain-containing proteins might lead to a plant-specific SMC5/6 cofactor mediating interaction with DNA repair complexes and/or chromatin.

Via two BLASTs, first against the moss *Physcomitrium patens* and then Arabidopsis, we found in total six Arabidopsis BRCT5 domain-containing proteins, including two already known DNA damage repair factors BRCA1 and BARD1. BRCA1 is a well-known tumor suppressor in humans that is evolutionarily conserved also in plants (Trapp et al., 2011). Studies in mammals and Arabidopsis revealed that BRCA1 and BARD1 frequently act as a heterodimer (Wu et al., 1996; Reidt et al., 2006). In Arabidopsis, both BRCA1 and BARD1 are necessary for resistance to DNA damage and also for normal levels of somatic homologous recombination (Reidt et al., 2006). Furthermore, the function of BARD1 seems to go beyond the regulation of genome stability because BARD1 was found to suppress the expression of *WUSCHEL1*, a master regulator homeobox gene controlling the stem cell pool (Mayer et al., 1998), in the shoot apical meristems and thus contributing to the meristem normal growth and organization during plant development (Han et al., 2008). Besides the established role of these two proteins in plant DNA damage repair, their exact molecular functions, including the binding targets of BRCT5 domains, remain unknown.

The BCP2, BCP3, and BCP4 proteins carry a pair of BRCT domains only at their C-termini. On contrary, BCP1 bears an additional pair of BRCT domains also at the N-terminus. The BRCT5 domain of all Arabidopsis BCPs shows a conserved pattern of specific amino acids with different properties. Furthermore, *in silico*-based modeling revealed a conserved structure of this domain in plants relative to the fission yeast Brc1. The only non-BRCT domain identified in BCPs was an N-terminally positioned Gcn5-related N-acetyltransferase domain (Uniprot) in BCP2. It implies that BCP2 might contribute to chromatin relaxation and/or transcription. However, none of the BCPs repeated the repertoire of domains in *Sp*Brc1 and/or *Hs*NSE5, suggesting that they are not direct Arabidopsis homologs, and biochemical studies will have to be conducted to explore their potential relationship at the protein-protein interaction level. BCP1 shows possible homologies to the human proteins PTIP and TOPIP1B. However, a significant homology is present only over the BRCT domain regions. Based on this, we conclude that all four identified BCPs represent novel plant-specific BRCT5 domain-containing proteins.

An important step toward the functional characterization of the BCPs was their response to DNA damage. The most promising candidate in DNA damage sensitivity assays was *BCP1*, while *BCP2, BCP3*, and *BCP4* did not differ significantly from wild-type. However, analysis of additional mutant alleles for at least *BCP2* and *BCP3* is needed because the alleles tested in this study most likely do not represent loss of function mutants. The *bcp2-1* allele may even be a *BCP2* overexpressor line. BCP1 loss-of-function mutants were hypersensitive to DNA DSBs caused by bleocin, DNA-inter-strand crosslinks induced by MMC, and two types of DNA-protein crosslinks caused by zebularine and CPT. Hence, BCP1 emerged from our analyses as an important player in DNA repair of multiple types of DNA lesions, possibly through a mainstream DNA repair pathway. The possible role of BCP2, BCP3, and BCP4 in e.g. repair of other types of DNA damage is not excluded and should be a focus of future studies.

We made an exciting observation that BCP1 is transcriptionally upregulated in response to gamma-radiation and MMC treatments and that the activation is SOG1-dependent. SOG1 is a plant-specific transcription factor that is phosphorylated by ATM and ATR kinases and orchestrates downstream responses of the key set of genes involved in the maintenance of genome stability, including cell cycle and homologous recombination repair (Yoshiyama et al., 2013a; Yoshiyama, 2016; Ogita et al., 2018). Two recent studies defined the SOG1 consensus binding motif CTT(N)_7_AAG and found that SOG1 is physically binding to the cis-regulatory region of *BCP1* in Arabidopsis (Bourbousse et al., 2018; Ogita et al., 2018). Surprisingly, we did not find any such a motif in the region upstream of the *BCP1* transcription start site which suggests a presence of a non-canonical SOG1 binding motif in the *BCP1* promoter.

The absence of *BCP1* transcriptional upregulation in the *sog1* mutant background also clearly places *BCP1* downstream of SOG1 in the same DNA damage repair pathway. Although *BCP1* transcription is enhanced by DNA damage, it is not fully dependent on it. This is apparent from the expression of *BCP1* promoter in both somatic and floral meristems without any stress. Our analysis suggests that *BCP1* is activated to a basal level in SOG1-independent and induced-DNA damage-independent manner. Whether this represents an activation induced by spontaneously occurring DNA damage (e.g. during DNA replication) remains to be studied. In summary, we identify *BCP1* as an Arabidopsis BRCT5 domain-containing gene directly transcriptionally controlled by SOG1 during induced DNA damage.

The critical experiment was the analysis of somatic homologous recombination using genetically engineered HR trap lines. This experiment showed a significantly reduced frequency of HR in *bcp1* mutant plants, strongly suggesting that BCP1 is needed for normal levels of HR. How BCP1 directly functions in this process is currently unknown. By its N- and C-terminal BRCT domains, it could bind two phosphorylated proteins and this way facilitate HR. Such interactors will be identified in the follow-up research.

In conclusion, out of four uncharacterized Arabidopsis BRCT5 domain-containing proteins, we identified BCP1 as a new Arabidopsis DNA damage repair factor that is directly controlled by SOG1 and ensures normal levels of homologous recombination.

## Data availability statement

The datasets presented in this study can be found in online repositories. The names of the repository/repositories and accession number(s) can be found below: https://www.ncbi.nlm.nih.gov/geo/, GSE112773.

## Author contributions

AP, JP, JD, and JV conceived and designed the study. JV, FY, and ET performed experiments. All authors analyzed data and interpreted the results. AP and JV wrote the paper. All authors contributed to the article and approved the submitted version.

## References

[B1] Alonso J. M. Stepanova A. N. Leisse T. J. Kim C. J. Chen H. Shinn P. . (2003). Genome-wide insertional mutagenesis of arabidopsis thaliana. Science 301, 653–657. doi: 10.1126/science.1086391 12893945

[B2] Altschul S. F. Gish W. Miller W. Myers E. W. Lipman D. J. (1990). Basic local alignment search tool. J. Mol. Biol. 215, 403–410. doi: 10.1016/S0022-2836(05)80360-2 2231712

[B3] Baubec T. Pecinka A. Rozhon W. Mittelsten Scheid O. (2009). Effective, homogeneous and transient interference with cytosine methylation in plant genomic DNA by zebularine. Plant J. 57, 542–554. doi: 10.1111/j.1365-313X.2008.03699.x 18826433 PMC2667684

[B4] Bork P. Hofmann K. Bucher P. Neuwald A. F. Altschul S. F. Koonin E. V. (1997). A superfamily of conserved domains in DNA damage- responsive cell cycle checkpoint proteins. FASEB J. 11, 68–76. doi: 10.1096/fasebj.11.1.9034168 9034168

[B5] Bourbousse C. Vegesna N. Law J. A. (2018). SOG1 activator and MYB3R repressors regulate a complex DNA damage network in arabidopsis. Proc. Natl. Acad. Sci. U.S.A. 115, E12453–E12462. doi: 10.1073/pnas.1810582115 30541889 PMC6310815

[B6] Chatterjee N. Walker G. C. (2017). Mechanisms of DNA damage, repair, and mutagenesis. Environ. Mol. Mutagen. 58, 235–263. doi: 10.1002/em.22087 28485537 PMC5474181

[B7] Hafner A. Bulyk M. L. Jambhekar A. Lahav G. (2019). The multiple mechanisms that regulate p53 activity and cell fate. Nat. Rev. Mol. Cell Biol. 20, 199–210. doi: 10.1038/s41580-019-0110-x 30824861

[B8] Han P. Li Q. Zhu Y.-X. (2008). Mutation of arabidopsis BARD1 causes meristem defects by failing to confine WUSCHEL expression to the organizing center. Plant Cell 20, 1482–1493. doi: 10.1105/tpc.108.058867 18591352 PMC2483370

[B9] Heyer W.-D. Ehmsen K. T. Liu J. (2010). Regulation of homologous recombination in eukaryotes. Annu. Rev. Genet. 44, 113–139. doi: 10.1146/annurev-genet-051710-150955 20690856 PMC4114321

[B10] Hu Z. Cools T. De Veylder L. (2016). Mechanisms used by plants to cope with DNA damage. Annu. Rev. Plant Biol. 67, 439–462. doi: 10.1146/annurev-arplant-043015-111902 26653616

[B11] Kleinboelting N. Huep G. Kloetgen A. Viehoever P. Weisshaar B. (2012). GABI-kat SimpleSearch: new features of the arabidopsis thaliana T-DNA mutant database. Nucleic Acids Res. 40, D1211–D1215. doi: 10.1093/nar/gkr1047 22080561 PMC3245140

[B12] Klepikova A. V. Kasianov A. S. Gerasimov E. S. Logacheva M. D. Penin A. A. (2016). A high resolution map of the arabidopsis thaliana developmental transcriptome based on RNA-seq profiling. Plant J. 88, 1058–1070. doi: 10.1111/tpj.13312 27549386

[B13] Lafarge S. Montané M.-H. (2003). Characterization of arabidopsis thaliana ortholog of the human breast cancer susceptibility gene 1: AtBRCA1 , strongly induced by gamma rays. Nucleic Acids Res. 31, 1148–1155. doi: 10.1093/nar/gkg202 12582233 PMC150221

[B14] Leung G. P. Lee L. Schmidt T. I. Shirahige K. Kobor M. S. (2011). Rtt107 is required for recruitment of the SMC5/6 complex to DNA double strand breaks. J. Biol. Chem. 286, 26250–26257. doi: 10.1074/jbc.M111.235200 21642432 PMC3138301

[B15] Li X. Liu K. Li F. Wang J. Huang H. Wu J. . (2012). Structure of c-terminal tandem BRCT repeats of Rtt107 protein reveals critical role in interaction with phosphorylated histone H2A during DNA damage repair. J. Biol. Chem. 287, 9137–9146. doi: 10.1074/jbc.M111.311860 22262834 PMC3308795

[B16] Lobet G. Pagès L. Draye X. (2011). A novel image-analysis toolbox enabling quantitative analysis of root system architecture. Plant Physiol. 157, 29–39. doi: 10.1104/pp.111.179895 21771915 PMC3165877

[B17] Manova V. Gruszka D. (2015). DNA Damage and repair in plants – from models to crops. Front. Plant Sci. 6. doi: 10.3389/fpls.2015.00885 PMC4617055 26557130

[B18] Mayer K. F. X. Schoof H. Haecker A. Lenhard M. Jürgens G. Laux T. (1998). Role of WUSCHEL in regulating stem cell fate in the arabidopsis shoot meristem. Cell 95, 805–815. doi: 10.1016/S0092-8674(00)81703-1 9865698

[B19] Menges M. Hennig L. Gruissem W. Murray J. A. H. (2002). Cell cycle-regulated gene expression inArabidopsis *. J. Biol. Chem. 277, 41987–42002. doi: 10.1074/jbc.M207570200 12169696

[B20] Molinier J. Ries G. Bonhoeffer S. Hohn B. (2004). Interchromatid and interhomolog recombination in arabidopsis thaliana. Plant Cell 16, 342–352. doi: 10.1105/tpc.019042 14729918 PMC341908

[B21] Nisa M.-U. Huang Y. Benhamed M. Raynaud C. (2019). The plant DNA damage response: signaling pathways leading to growth inhibition and putative role in response to stress conditions. Front. Plant Sci. 10. doi: 10.3389/fpls.2019.00653 PMC6534066 31164899

[B22] Ogita N. Okushima Y. Tokizawa M. Yamamoto Y. Y. Tanaka M. Seki M. . (2018). Identifying the target genes of SUPPRESSOR OF GAMMA RESPONSE 1, a master transcription factor controlling DNA damage response in arabidopsis. Plant J. 94, 439–453. doi: 10.1111/tpj.13866 29430765

[B23] Oravcová M. Gadaleta M. C. Nie M. Reubens M. C. Limbo O. Russell P. . (2019)Brc1 promotes the focal accumulation and SUMO ligase activity of Smc5-Smc6 during replication stress. In: Molecular and cellular biology. Available at: https://journals.asm.org/doi/full/10.1128/MCB.00271-18 (Accessed May 11, 2022). 10.1128/MCB.00271-18 PMC6321882 30348841

[B24] Orel N. Kyryk A. Puchta H. (2003). Different pathways of homologous recombination are used for the repair of double-strand breaks within tandemly arranged sequences in the plant genome. Plant J. 35, 604–612. doi: 10.1046/j.1365-313X.2003.01832.x 12940953

[B25] Preuss S. B. Britt A. B. (2003). A DNA-damage-induced cell cycle checkpoint in arabidopsis. Genetics 164, 323–334. doi: 10.1093/genetics/164.1.323 12750343 PMC1462560

[B26] Prochazkova K. Finke A. Tomaštíková E. D. Filo J. Bente H. Dvořák P. . (2022). Zebularine induces enzymatic DNA–protein crosslinks in 45S rDNA heterochromatin of arabidopsis nuclei. Nucleic Acids Res. 50, 244–258. doi: 10.1093/nar/gkab1218 34904670 PMC8754632

[B27] Puchta H. Swoboda P. Hohn B. (1995). Induction of intrachromosomal homologous recombination in whole plants. Plant J. 7, 203–210. doi: 10.1046/j.1365-313X.1995.7020203.x

[B28] R Core Team . (2018). R: A Language and Environment for Statistical Computing (Vienna:R Foundation for Statistical Computing). Available at: https://www.R-project.org.

[B29] Räschle M. Smeenk G. Hansen R. K. Temu T. Oka Y. Hein M. Y. . (2015). Proteomics reveals dynamic assembly of repair complexes during bypass of DNA cross-links. Science 348, 1253671. doi: 10.1126/science.1253671 25931565 PMC5331883

[B30] Razqallah H. (2008). DNA-Damage repair; the good, the bad, and the ugly. EMBO J. 27, 589–605. doi: 10.1038/emboj.2008.15 18285820 PMC2262034

[B31] Reidt W. Wurz R. Wanieck K. Ha Chu H. Puchta H. (2006). A homologue of the breast cancer-associated gene BARD1 is involved in DNA repair in plants. EMBO J. 25, 4326–4337. doi: 10.1038/sj.emboj.7601313 16957774 PMC1570427

[B32] Seton-Rogers S. (2006). Putting p53 in context. Nat. Rev. Cancer 6, 423–423. doi: 10.1038/nrc1924

[B33] Shultz R. W. Tatineni V. M. Hanley-Bowdoin L. Thompson W. F. (2007). Genome-wide analysis of the core DNA replication machinery in the higher plants arabidopsis and rice. Plant Physiol. 144, 1697–1714. doi: 10.1104/pp.107.101105 17556508 PMC1949880

[B34] Swoboda P. Gal S. Hohn B. Puchta H. (1994). Intrachromosomal homologous recombination in whole plants. EMBO J. 13, 484–489. doi: 10.1002/j.1460-2075.1994.tb06283.x 8313893 PMC394832

[B35] Trapp O. Seeliger K. Puchta H. (2011). Homologs of breast cancer genes in plants. Front. Plant Sci. 2. doi: 10.3389/fpls.2011.00019 PMC3355568 22629260

[B36] Varadi M. Anyango S. Deshpande M. Nair S. Natassia C. Yordanova G. . (2022). AlphaFold protein structure database: massively expanding the structural coverage of protein-sequence space with high-accuracy models. Nucleic Acids Res. 50, D439–D444. doi: 10.1093/nar/gkab1061 34791371 PMC8728224

[B37] Wan B. Hang L. E. Zhao X. (2016). Multi-BRCT scaffolds use distinct strategies to support genome maintenance. Cell Cycle 15, 2561–2570. doi: 10.1080/15384101.2016.1218102 27580271 PMC5053572

[B38] Williams J. S. Williams R. S. Dovey C. L. Guenther G. Tainer J. A. Russell P. (2010). gammaH2A binds Brc1 to maintain genome integrity during s-phase. EMBO J. 29, 1136–1148. doi: 10.1038/emboj.2009.413 20094029 PMC2845269

[B39] Wuest S. E. Vijverberg K. Schmidt A. Weiss M. Gheyselinck J. Lohr M. . (2010). Arabidopsis female gametophyte gene expression map reveals similarities between plant and animal gametes. Curr. Biol. 20, 506–512. doi: 10.1016/j.cub.2010.01.051 20226671

[B40] Wu L. C. Wang Z. W. Tsan J. T. Spillman M. A. Phung A. Xu X. L. . (1996). Identification of a RING protein that can interact *in vivo* with the BRCA1 gene product. Nat. Genet. 14, 430–440. doi: 10.1038/ng1296-430 8944023

[B41] Yan W. Shao Z. Li F. Niu L. Shi Y. Teng M. . (2011). Structural basis of γH2AX recognition by human PTIP BRCT5-BRCT6 domains in the DNA damage response pathway. FEBS Lett. 585, 3874–3879. doi: 10.1016/j.febslet.2011.10.045 22064073

[B42] Yan S. Wang W. Marqués J. Mohan R. Saleh A. Durrant W. E. . (2013). Salicylic acid activates DNA damage responses to potentiate plant immunity. Mol. Cell 52, 602–610. doi: 10.1016/j.molcel.2013.09.019 24207055 PMC3863363

[B43] Yoshiyama K. O. (2016). SOG1: a master regulator of the DNA damage response in plants. Genes Genet. Syst. 90, 209–216. doi: 10.1266/ggs.15-00011 26617076

[B44] Yoshiyama K. Conklin P. A. Huefner N. D. Britt A. B. (2009). Suppressor of gamma response 1 (SOG1) encodes a putative transcription factor governing multiple responses to DNA damage. Proc. Natl. Acad. Sci. 106, 12843–12848. doi: 10.1073/pnas.0810304106 19549833 PMC2722309

[B45] Yoshiyama K. Kobayashi J. Nobuo O. Minako U. Kimura S. Maki H. . (2013a). ATM-Mediated phosphorylation of SOG1 is essential for the DNA damage response in arabidopsis. EMBO Rep. 14, 817–822. doi: 10.1038/embor.2013.112 23907539 PMC3790055

[B46] Yoshiyama K. O. Sakaguchi K. Kimura S. (2013b). DNA Damage response in plants: conserved and variable response compared to animals. Biology 2, 1338–1356. doi: 10.3390/biology2041338 24833228 PMC4009792

[B47] Yu X. Chini C. C. S. He M. Mer G. Chen J. (2003). The BRCT domain is a phospho-protein binding domain. Science 302, 639–642. doi: 10.1126/science.1088753 14576433

[B48] Zhang X. Henriques R. Lin S.-S. Niu Q.-W. Chua N.-H. (2006). Agrobacterium-mediated transformation of arabidopsis thaliana using the floral dip method. Nat. Protoc. 1, 641–646. doi: 10.1038/nprot.2006.97 17406292

