# Supplementary material for: Analysis of BRCT5 domain-containing proteins reveals a new component of DNA damage repair in Arabidopsis
Source: Front Plant Sci. 2022 Dec 12;13:1023358. doi: 10.3389/fpls.2022.1023358 (PMC9791218; doi:10.3389/fpls.2022.1023358)
Supplement: Supplementary file 1 [file DataSheet_1.pdf]

Supplementary materials

Analysis of BRCT5 domain containing proteins reveals a new component of DNA damage repair in Arabidopsis

Jovanka Vladejić[1], Fen Yang[1,2], Eva Dvořák Tomaštíková[1], Jaroslav Doležel[1], Jan J. Palecek[3], Ales Pecinka[1,2,*]

[1] Institute of Experimental Botany (IEB), Czech Acad Sci, Centre of the Region Haná for Biotechnological and Agricultural Research (CRH), Šlechtitelů 31, 77900 Olomouc, Czech Republic, [2] Department of Cell Biology and Genetics, Faculty of Science, Palacký University, Šlechtitelů 27, 77900 Olomouc, Czech Republic, [3] National Centre for Biomolecular Research (NCBR), Faculty of Science, Masaryk University, Kamenice 5, 62500 Brno, Czech Republic

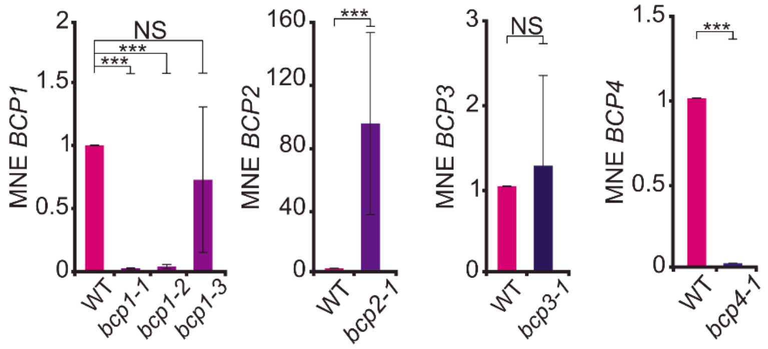

Supplementary Figure 1 | Expression of BCP genes in the respective *bcp* mutants. Reverse transcription qPCR analysis of *BCP1*, *BCP2*, *BCP3* and *BCP4* expression in wild-type (WT) and respective *bcp* mutant plants under ambient conditions. Y-axis shows mean normalized expression relative to *PP2A*. Error bars show three biological replicates. NS = not significantly different, *** P < 0.001 statistical difference in Mann-Whitney U-test test at P < 0.05.

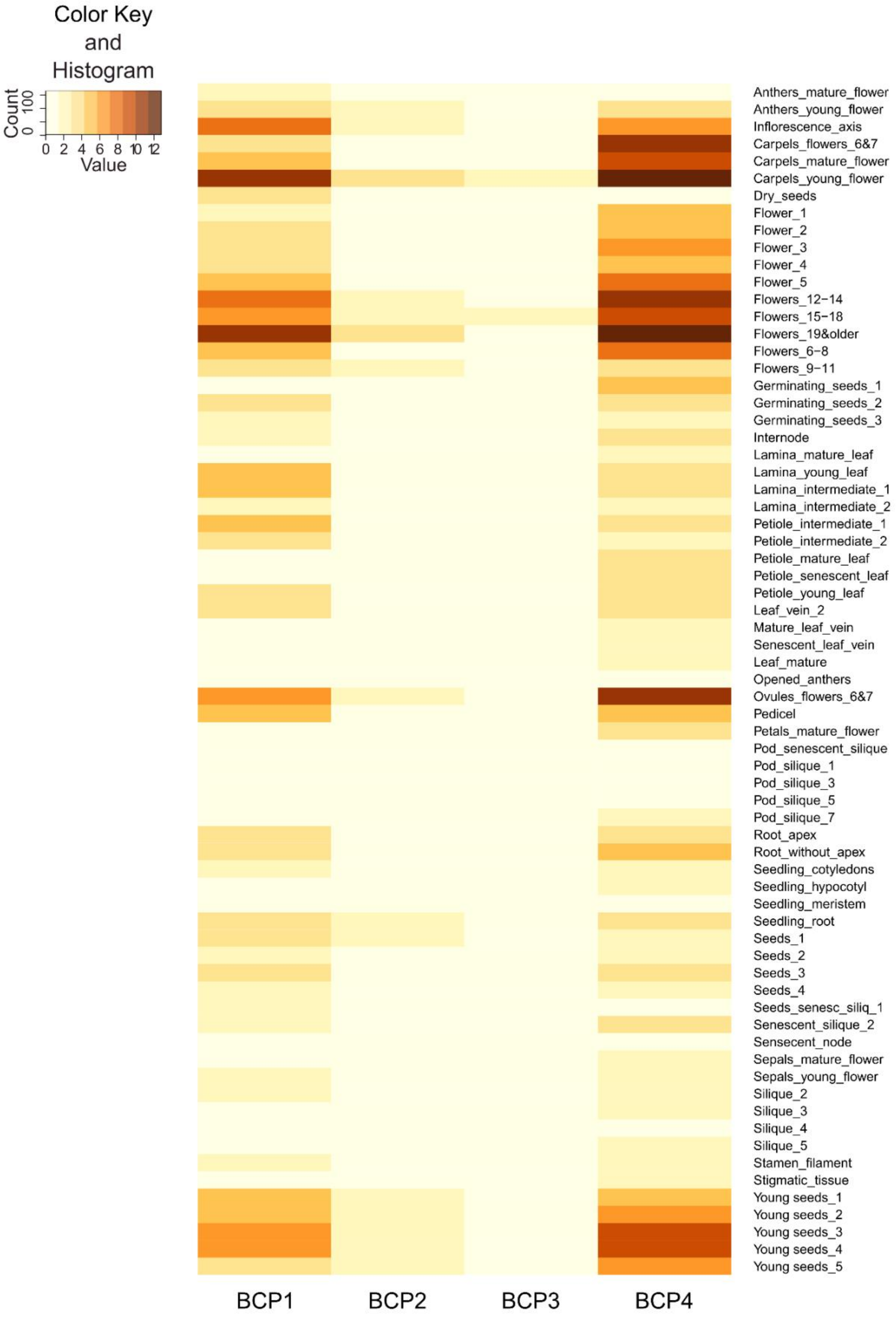

Supplementary Figure 2 | Expression of BCP genes during plant development. The expression values for individual genes on x-axis correspond to number of fragments per kilobase per million (FPKM). The y-axis shows individual tissues. The expression values were retrieved from a public dataset (Klepikova et al., 2016).

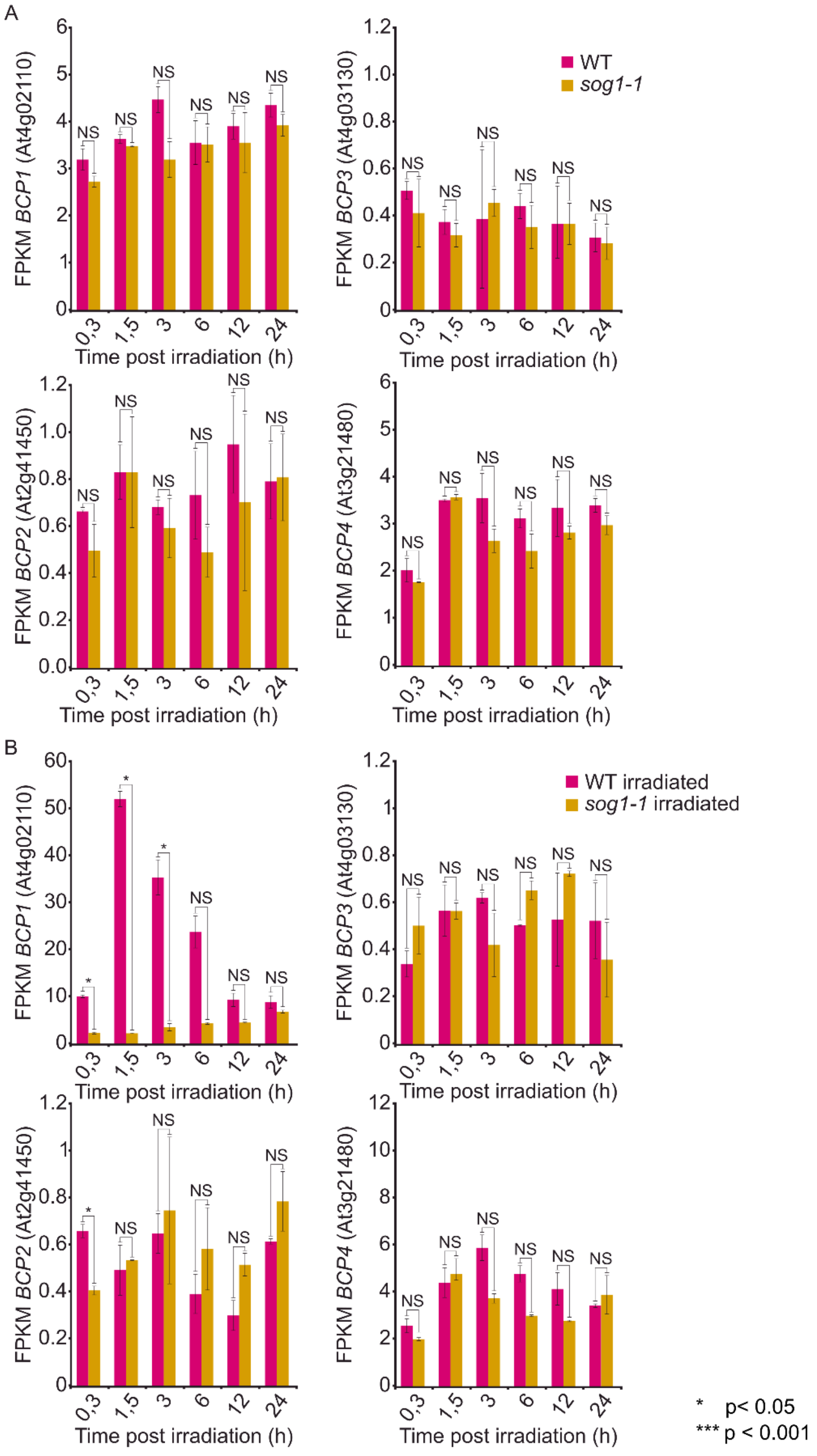

Supplementary Figure 3 | (**A**) Expression of BCP genes under mock conditions in wild-type and *sog1-1* plants. (**B**) Expression of BCP genes after gamma irradiation in wild type and *sog1-1* plants. The x-axis shows different sampling times after gamma irradiation (hours) and the y-axis shows a number of fragments per kilobase per million (FPKM). Asterisks represent significant differences in two sample T-test with unequal variance, * P < 0.05, *** P < 0.001, NS – not significantly different. The expression values were extracted from the public dataset (Bourbousse et al., 2018).

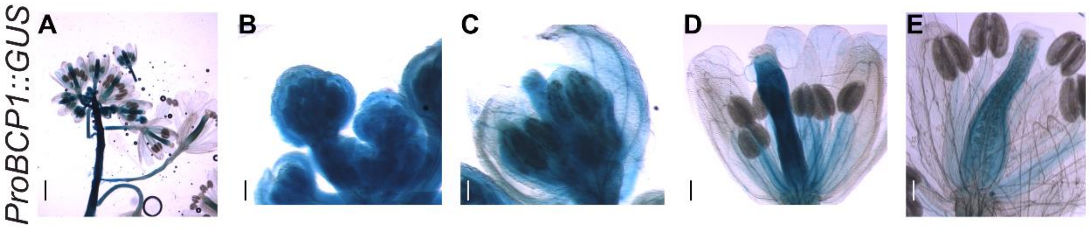

Supplementary Figure 4 | *In planta* analysis of *BCP1* expression in flower organs. Stable transformants carrying *ProBCP1::GUS* were grown in soil until flowering. Expression of *BCP1* was monitored after GUS histochemical staining of the inflorescences. Representative pictures showing the tissues with *BCP1* expression. (**A**) Full inflorescence. Scale bar = 1 mm. (**B**) Closed young flowers. Scale bar = 100 µm. (**C**) Young open flower. Scale bar = 200 µm. (**D**) Open flower with anthers and pistil. Scale bar = 100 µm. (**E**) Old flower with anthers and pistil. Scale bar = 200 µm.

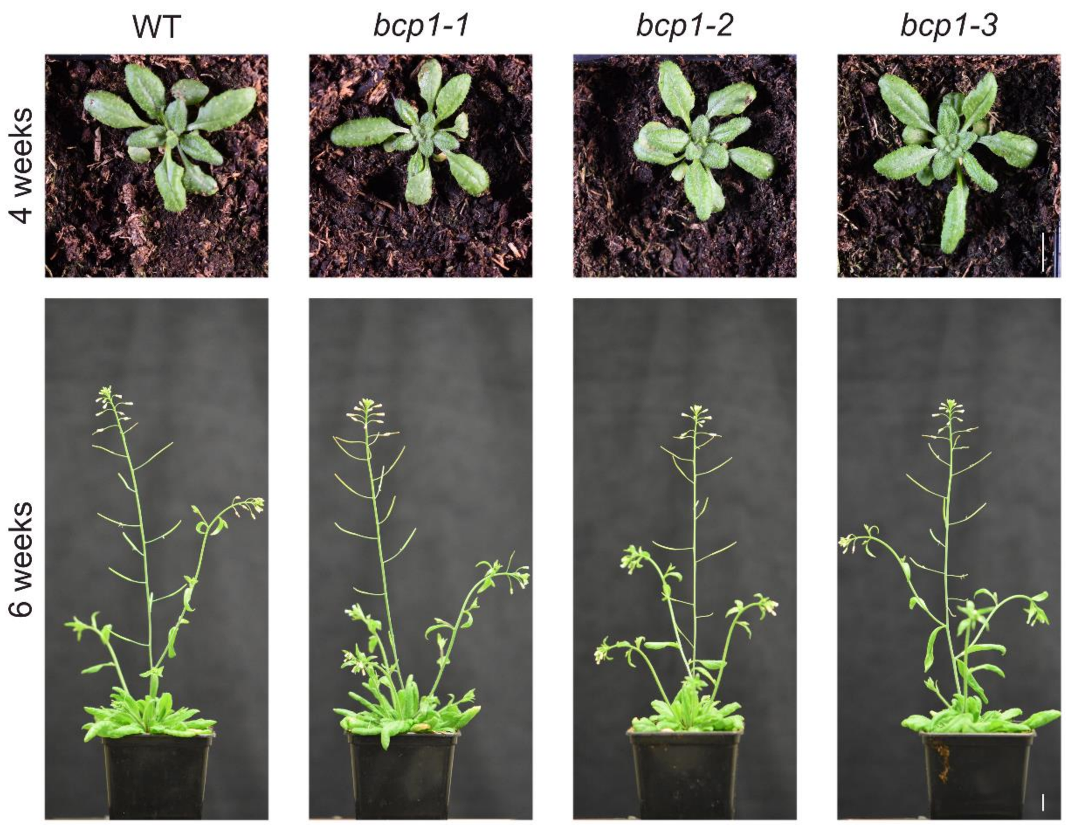

Supplementary Figure 5 | Phenotypes of *BCP* mutant plants grown in soil. Homozygous BCP mutants were grown in soil and photographed at the rosette stage (4 weeks) and at seed setting (8 weeks). Scale bar = 1 cm.

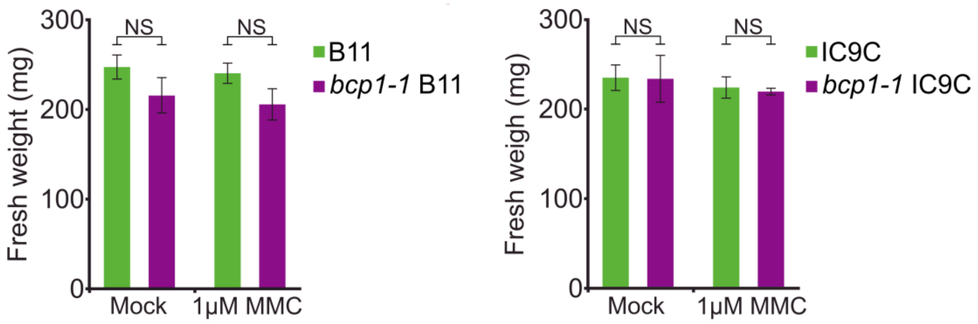

Supplementary Figure 6 | Fresh weight measurements as the control for HR assays show no significant difference between control marker lines B11 and IC9C and marker lines in bcp1-1 background. B11, *bcp1-1* B11, IC9C and *bcp1-1* IC9C seedlings were grown for 10 days on ½ MS (MOCK) or ½ MS with 1 µM MMC (MMC). Three replicates, consisting of 60 plants each, were measured for each group. The statistical significance of data was assessed by Mann-Whitney U-test. NS – not significantly different.

Gene	Name	Sequence (5' to 3')	Use
At4g02110	*bcp1-1*_LP	TGTATTAGTGGACGCCTGGAATTG	genotyping
At4g02110	*bcp1-1*_RP	AGTGTTTAACTCACTCGTGGGTGA	genotyping
At4g02110	*bcp1-2*_LP	GATGGTCTTTCTCTTCTGGGG	genotyping
At4g02110	*bcp1-2*_RP	CGCCAGAGACTGATACTTTGG	genotyping
At4g02110	*bcp1-3*_LP	AGATTTGAATGGGATTCCAGG	genotyping
At4g02110	*bcp1-3*_RP	CCAAAGTATCAGTCTCTGGCG	genotyping
At2g41450	*bcp2-1*_LP	TTTGGGTCGGATTCGGGATTTTT	genotyping
At2g41450	*bcp2-1*_RP	AGTTGACAACTTGAACGTTTGTTAC	genotyping
At4g03130	*bcp3-1*_LP	CACGCATCAAATCTAGCCAAG	genotyping
At4g03130	*bcp3-1*_RP	ATCTTCAATTTCCCCACATCC	genotyping
At3g21480	*bcp4-1*_LP	CTGCCTTGCATTCTTTTCAAG	genotyping
At3g21480	*bcp4-1*_RP	TGTAAGACAACTCGCCTCACC	genotyping
o8474		ATAATAACGCTGCGGACATCTACATTTT	genotyping
LBb1.3		ATTTTGCCGATTTCGGAAC	genotyping
At4g02110	*proBCP1*_FWD	AAAAAGCAGGCTTATTAAAAATTTGTAAGTAA AACCATTTGCTATAACAAGAATTTATAGCT	cloning
At4g02110	*proBCP1*_REV	AGAAAGCTGGGTTTTTTTTTTTTGAAAAATTAG GGTTTTATTAGGGTGGAGG	cloning
At4g02110	attB1 adapter	GGGGACAAGTTTGTACAAAAAAGCAGGCT	cloning
At4g02110	attB2 adapter	GGGGACCACTTTGTACAAGAAAGCTGGGT	cloning
At4g02110	*BCP1_FWD*	TGCAGAGGTGGAAATTACGGTGCTAG	RT-qPCR
At4g02110	*BCP1_REV*	TTTACCTACACCAGCCTCCCTTTTGC	RT-qPCR
At2g41450	*BCP2_FWD*	TTCATTGGTTTTGAAGTCCACGCTTG	RT-qPCR
At2g41450	*BCP2_REV*	GTGTGTATATGTTACAGCAGCAAGAGGT	RT-qPCR
At4g03130	*BCP3_FWD*	CCCCATTTCAAGTGCTCTACGACGAT	RT-qPCR
At4g03130	*BCP3_REV*	AGCAGCGACACTTCCATCTTCATCAT	RT-qPCR
At3g21480	*BCP4_FWD*	TGGGCTCGTTCTGATTCCAAACTGTT	RT-qPCR
At3g21480	*BCP4_REV*	ATCATTCCCTAAAACTGCCGCTTCAC	RT-qPCR
At1g69960	*PP2A_FWD*	TAACGTGGCCAAAATGATGC	RT-qPCR
At1g69960	*PP2A_REV*	GTTCTCCACAACCGCTTGGT	RT-qPCR

Supplementary Table 1 | Oligonucleotides used in this study.
